# Generational differences in patterns of physical activities over time in the Canadian population: an age-period-cohort analysis

**DOI:** 10.1186/s12889-018-5189-z

**Published:** 2018-03-02

**Authors:** Mayilee Canizares, Elizabeth M. Badley

**Affiliations:** 1 The Arthritis Program. Krembil Research Institute, University Health Network, 399 Bathurst St, MP10-316, Toronto, ON M5T 2S8 Canada; 2 Arthritis Community Research and Evaluation Unit. Krembil Research Institute, University Health Network, 399 Bathurst St, MP10-310, Toronto, ON M5T 2S8 Canada

**Keywords:** Leisure time physical activity, Active commuting, Sedentary behavior, Birth cohort, Period effect

## Abstract

**Background:**

Using longitudinal panel data, the aim of this study was to examine the contribution of age, period, and cohort effects on changes in physical activity over time in a population-based sample of Canadians. We focused on three domains of physical activities: leisure time, commuting (i.e. walking and cycling), and daily activities (i.e. sedentary behavior). We also examined whether changes in sedentary behavior related to changes in participation in leisure time and commuting activities.

**Methods:**

We used data from the Longitudinal National Population Health Survey (1994–2011): 10050 participants born between 1935 and 1984 grouped in five 10-years birth cohorts. We examined three outcomes: moderate-to-vigorous leisure time physical activity, active commuting, and sedentary behavior. We also included education, income, and body mass index as covariates. We used hierarchical age-period-cohort analysis to examine the contribution of age, period, and cohort effects to changes over time for each outcome.

**Results:**

We found that recent cohorts were more likely to report sedentary behavior and greater participation in leisure time physical activities and active commuting. We also found a significant trend of increasing participation in active leisure time physical activity and active commuting among Canadians from 1994/95 to 2010/11 and, at the same time, an increase in sedentary behavior. The greater participation in leisure time physical activities and active commuting in each succeeding recent cohort was partially related to the secular trend of increasing participation in physical activities over time in the population. Furthermore, those with sedentary behavior were less likely to report participation in physical activities. Overall, obese individuals were less likely to be physically active and more likely to be sedentary, while the effect of socio-economic status varied by outcome.

**Conclusions:**

The greater participation in physical activities (leisure time and commuting) in recent cohorts is encouraging and was substantially explained by period effects, which reflect broad social and environmental factors affecting the whole population. The large cohort effect of increasing sedentary behavior and the inverse relationship between sedentary behavior and physical activity is concerning, and identifies a target group for future interventions.

**Electronic supplementary material:**

The online version of this article (10.1186/s12889-018-5189-z) contains supplementary material, which is available to authorized users.

## Background

There is compelling evidence of the positive effects of physical activity on health [1, 2]. However, it has been reported that a large proportion of the population do not achieve recommended physical activity levels [3, 4], even when active commuting (i.e. walking and cycling), an important contributor to overall physical activity levels among young and middle-aged adults [5], is taken into account. There is also emerging evidence that time spent in sedentary activities is a risk factor for chronic conditions, such as diabetes and cardiovascular diseases [6, 7] and premature mortality [8, 9], irrespective of the overall physical activity levels. Sedentary activities are those with low energy expenditure, such as prolong sitting time at work or at home, sleeping, lying down, and watching television [10].

Previous studies examining changes in physical activity over time have mainly focused on time trends in leisure time physical activities (LTPA) such as exercise, sports, and gardening [11–20] and less attention have been given to time trends in active commuting and sedentary daily activities [16, 17, 19, 21, 22]. US data suggest that participation in LTPA has declined or remained stable over time [15]. In contrast, studies from Canada, Denmark, and Finland suggest that participation in LTPA have increased in more recent years [17–20]. Few studies have examined changes in active commuting also showing variable results [17, 19, 22]. A study found that participation in active commuting among adults in Finland declined from the 1970s to the early 2000s [17]. In contrast, in the same period of time, active commuting increased among Canadian adults [19]. A US study comparing data from 2001 to 2009 found that the overall prevalence of active commuting was low and that there were modest increases in walking while cycling levels were stable [22]. The studies that have examined changes in sedentary daily activities have reported an increase in sedentary time spent at work or at home in recent years across all jurisdictions [15, 17, 19, 23].

Variations in physical activity over time may be related to the effects of aging, to the different life experiences of generations of people born at different times (cohort effects), or these variations could also be the result of societal and environmental changes which affect the population as a whole (period effects). Only few studies have examined age, period, and cohort effects in LTPA [16, 17, 24–26], active commuting [17], and daily activity [17, 26]. Given this gap in the literature, the goals of this paper are: 1) to examine age, period, and cohort effects in physical activity across three domains: leisure time, commuting, and sedentary behavior (sedentary time spent at work or at home) over 16 years in a representative sample of Canadians, 2) to examine whether age, period, and cohort effects are explained by changes in education, income, and body mass index (BMI), and 3) to examine whether changes in sedentary behavior over time influence the trajectories of participation in LTPA and active commuting.

## Methods

### Data source

This study used data from 1994/95 to 2010/11 of the longitudinal component of the National Population Health Survey (NPHS), a representative sample of the Canadian population. The target population of the survey included household residents in the ten Canadian provinces in 1994/1995 (cycle 1) excluding persons living on Indian Reserves and Crown Lands, residents of health institutions, full-time members of the Canadian Forces Bases and some remote areas in Ontario and Quebec. The longitudinal NPHS retained individuals who moved to long-term care institutions and those who died over the course of the survey. The death of a respondent was confirmed against the Canadian Vital Statistics Database, and the cause and date of death were captured. More detailed descriptions of the NPHS design and interview procedures are available from Statistics Canada [27]. The NPHS longitudinal sample included 17,276 participants from all ages in 1994/1995 and these same participants were interviewed every 2 years. The present study included 12,016 participants who were born between 1935 and 1984 (aged 10–59 years in 1994/95). We excluded 1966 participants who provided data in 1 or 2 cycles only (*n* = 1234) and those with incomplete data on the physical activity variables at baseline (*n* = 732). This resulted in a sample of 10,050 participants for analysis. We compared baseline characteristics (age, sex, education, income, BMI, and physical activity variables) between those included and excluded. We found that those excluded were more likely to be older, with lower income, and had lower BMI. In addition, no significant differences related to the physical activity variables were seen.

### Outcomes

#### Active LTPA

Participants answered a series of questions about their participation in various LTPA such as walking for exercise, running, and gardening. Participants reported how many times they performed each activity over the past 3 months and the average duration of each session. Daily energy expenditure was calculated for each activity using the general methodology described by Katzmarzyk and Tremblay [28]. Participants were categorized as having active LTPA (≥1.5 kcal/ kg/day) versus inactive (no physical activity to less than 1.5 kcal/ kg/ day) based on their total energy expenditure. This categorization is equivalent to walking at least 30 min every day [28].

#### Active commuting

Participants were asked “In a typical week, how much time did you usually spend walking (bicycling) to work or to school or while doing errands?” Response options were: “None”, “< 1 h”, “1–5 h”, “6–10 h”, “11–20 h”, “> 20 h”. Those reporting 6 h or more were considered to participate in active commuting.

#### Sedentary behavior

Participants were asked “Thinking back over the past 3 months, which of the following best describes your usual daily activities or work habits?” Response options were: “Usually sit during the day and don’t walk around very much”, “Stand or walk quite a lot during the day but don’t have to carry or lift things very often”, “Usually lift or carry light loads, or have to climb stairs or hills often”, and “Do heavy work or carry very heavy loads.” Respondents who answered “usually sit during the day and don’t walk around very much” were considered having a sedentary behavior.

### Covariates

#### Age, period, and cohort

We allocated participants into five cohorts according to their birth date: 1940s cohort (born: 1935–1944), 1950s (born: 1945–1954), 1960s (born: 1955–1964), 1970s (born: 1965–1974), and 1980s (born: 1975–1984). At each cycle, the age (in years) was calculated and survey year was used as indicator of period.

#### Individual variables

In addition to the three time-related variables —age, period, and cohort— we also included sex, education, income, and body mass index (BMI). The number of years of schooling was used as a measure of educational attainment and grouped as: less than 12 years, 12–15 years, and 16 years or above. Household income was grouped in quartiles of the overall distribution and we kept non-response in a separate category for analysis. BMI was calculated from self-reported weight and height and grouped into five categories: underweight (< 18.5), normal weight (18.5–24.9), overweight (25–29.9), moderately obese (30.0–34.9), and severe obese (≥35.0). These variables, with the exception of sex, were collected at each interview.

#### Statistical analysis

The analytical framework for this study is the same we used in our previous work: a hierarchical age-period-cohort (HAPC) modeling strategy to understand the contribution of age, period, and cohort to time changes in the outcomes [29, 30]. In the conceptualization of the model, observations are nested within individuals and individuals nested within time periods [31]. In the HAPC model, age and cohort were estimated as fixed effects while period was estimated as a random effect. For each outcome we started with a two-level growth model, with observations nested within individuals, and built complexity by adding period in the random part and the explanatory variables in the fixed part. The first model was a two-level growth model with fixed effects for age and cohort (Model 1). We examined linear and quadratic age terms and retained the best transformation. Then, we extended this two-level model to three levels to examine variation in the outcomes by period (Model 2). By comparing Model 2 to Model 1 we can examine whether any age and cohort effects are affected by period effects. We then added education, income, and BMI groups to examine the contribution of these explanatory variables to the age, period, and cohort effects (Model 3). For our third objective, sedentary behavior was added to the models for active LTPA and active commuting. In addition to examining the main effect for sedentary behavior we tested for interactions between sedentary behavior and age and sedentary behavior and birth cohort, in order to determine if the trajectories of active LTPA and active commuting differed for those with and without sedentary behavior (Model 4). In all models age was centered at 35 years (the median of the distribution for the five cohorts at baseline (1994/95)). Models were fit using the GLIMMIX procedure from SAS/STAT software including incomplete cases up to the point at which they drop-out or died and maximum likelihood estimators were used that adjust for non-response assuming the data are missing at random [32].

## Results

### Descriptive

There were 10,050 participants (aged 10 to 59 years) who met the inclusion criteria in 1994/95 with an average of 12 years of follow-up (maximum follow-up was 16 years): 1526 in the 1940s cohort, 2118 in the 1950s, 2997 in the 1960s, 2135 in the 1970s, and 1574 in the 1980s. The characteristics of the cohorts at baseline (1994/95) and at the end of the study (2010/11) are presented in Table 1. By 2010/11, about 35% of participants died or dropped-out. The main reasons for attrition were dropping-out the study and mortality particularly in the 1940s cohort. Active LTPA, active commuting, and sedentary behavior increased in all cohorts over the study period. However, physical activity levels were still low, in particular for active commuting. Also important to note is that more than half of those with active commuting were also active during leisure time (Data not shown).

**Table 1:** Characteristics of participants in each birth cohort^a^. Canadian National Population Health Survey, 1994–2011

	1940s(1935–1944)		1950s(1945–1954)		1960s(1955–1964)		1970s(1965–1974)		1980s(1975–1984)	
	Cycle 1 1994/95	Cycle 9 2010/11	Cycle 1 1994/95	Cycle 9 2010/11	Cycle 1 1994/95	Cycle 9 2010/11	Cycle 1 1994/95	Cycle 9 2010/11	Cycle 1 1994/95	Cycle 9 2010/11
Number in cohort	1526	989	2118	1529	2697	1841	2135	1333	1574	946
% active LTPA	34.1	54.9	37.2	53.8	37.9	54.6	43.7	57.6	47.8	60.6
% Active commuting	13.2	18.5	12.9	19.4	16.7	17.0	18.8	18.6	28.3	16.0
% Sedentary behavior	19.9	17.5	21.5	22.9	19.8	21.5	18.6	31.9	31.3	31.5
Mean Age	53.8	69.7	43.8	59.7	33.8	49.9	24.1	40.2	14.2	30.2
% Women	53.7	55.9	52.2	52.9	54.3	56.4	54.0	55.0	50.2	52.9
Mean years of schooling	11.6	12.1	12.9	13.3	13.2	13.7	13.3	14.1	8.9	14.6
Mean household income^a^	53.7	55.1	57.3	75.5	51.7	87.4	46.9	89.5	54.0	84.7
Mean BMI	26.7	27.4	26.0	27.8	25.2	27.6	24.4	27.2	21.1	26.4
Attrition^b^										
% Drop-out	18.7	–	22.4	–	29.0	–	36.3	–	40.8	–
% Dead	13.0	–	5.1	–	2.1	–	1.3	–	0.7	–

LTPA, Leisure Time Physical Activity; BMI, Body Mass Index

^a^In Canadian dollars. ^b^Proportion calculated out of sample at cycle 1

### Age-period-cohort effects

Results for modeling age-period-cohort effects are presented graphically with details of the full models presented in Additional files 1, 2, and 3 for active LTPA, active commuting, and sedentary behavior, respectively.

**Fig. 1:**
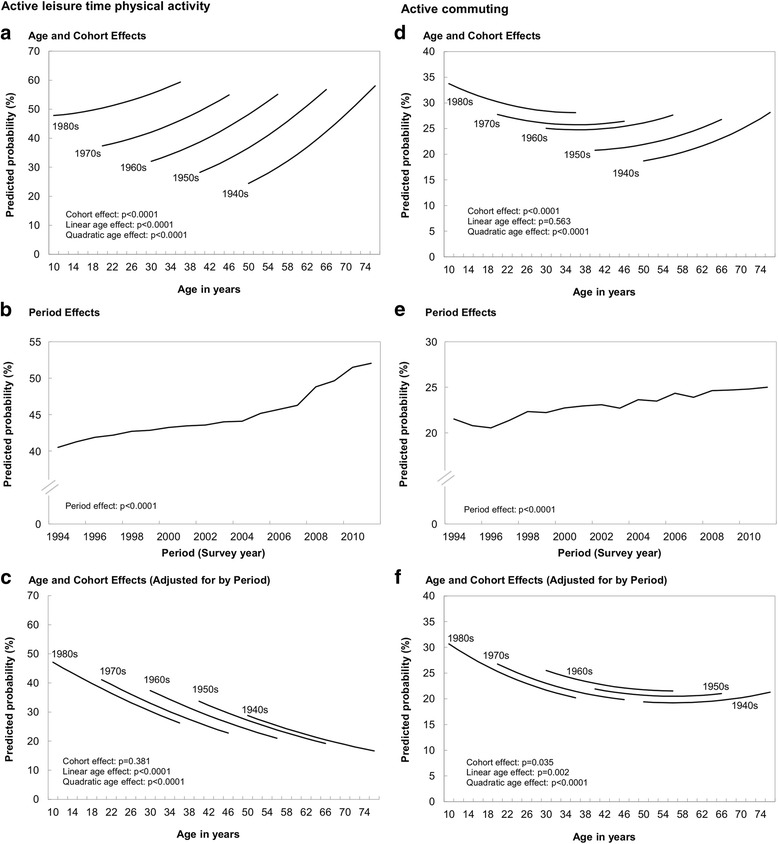
Age, Period, and Cohort effects on active leisure time physical activity and active commuting. Canadian National Population Health Survey, 1994–2011, Birth cohorts: 1980s = Born 1975–84; 1970s = Born 1965–74; 1960s = Born 1955–64; 1950s = Born 1945–55; 1940s = Born 1935–44. LTPA, leisure time physical activity. Values in A are predictions from model 1in Additional file 1 and values in B and C are predictions from model 2 in Additional file 1. Values in D are predictions from model 1in Additional file 2 and values in E and F are predictions from model 2 in Additional file 2. Each panel shows *p*-values for age, period, and/or cohort effects from the corresponding models

Fig. 1a shows the estimated age trajectory for participation in active LTPA for each cohort obtained from the model with age and cohort effects (Additional file 1, Model 1), unadjusted by period. There was a marked cohort effect with each succeeding recent birth cohort being more likely to report participating in active LTPA. In other words, comparing people at the same age indicate that those in more recent cohorts were more likely to report participating in active LTPA. There was an increasing age-trajectory of active LTPA indicating that people were more likely to report participating in active LTPA as they age in contrast to accepted understanding of a decline with age [33]. Fig. 1b shows the effect of period: the proportion reporting participating in LTPA increased steadily over the 16 years of the study. When the age-cohort model was adjusted for period (Fig. 1c, Model 2 in Additional file 1), cohort differences were no longer significant and the age-related increases in active LTPA were no longer seen. The proportion reporting active LTPA was now shown to decrease with age. Thus, comparing the findings from Model 2 with those from Model 1 tells us that the apparent increase with age and the greater participation in active LTPA seen in recent cohorts were largely due to a period effect of an overall increase in reporting active LTPA over time. Findings for active commuting are also presented in Fig. 1d, e, and f. Overall the findings were similar to those for active LTPA, although less marked. Recent cohorts were more likely to report active commuting (Fig. 1d), and there is likewise an increase in active commuting over time (Fig. 1E). When period was added to the age-cohort model, cohort differences were no longer significant (Fig. 1F).

**Fig. 2:**
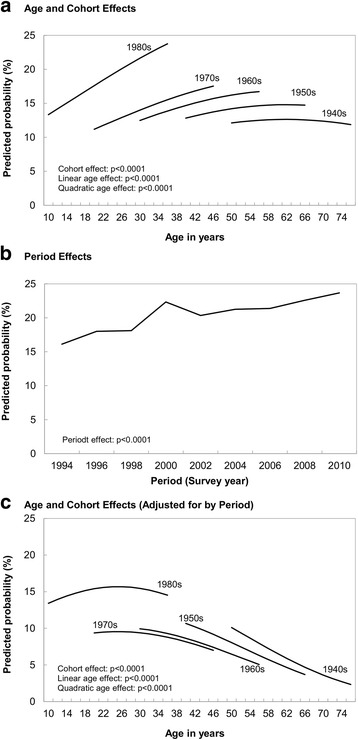
Age, period, and cohort effects on sedentary behavior. Canadian National Population Health Survey, 1994–2011 Birth cohorts: 1980s = Born 1975–84; 1970s = Born 1965–74; 1960s = Born 1955–64; 1950s = Born 1945–55; 1940s = Born 1935–44. Values in A are predictions from model 1 and values in B and C are predictions from model 2 in Additional file 3. Each panel shows p-values for age, period, and/or cohort effects from the corresponding models

The age-cohort model for sedentary behavior showed a similar pattern to that for active LTPA: There was a marked cohort effect with more recent cohorts being more likely to be sedentary and sedentary behavior peaked at middle age (Fig. 2a, Model 1 in Additional file 1). There was also indication of a significant period effect of an increase in sedentary behavior over time (Fig. 2b). After taking into account this period effect, the cohort differences were reduced but remained significant: at corresponding ages, those who were born in the 1960s and 1970s were less likely to be sedentary than those born in the 1980s, 1950s or 1940s (Fig. 2c).

### Sex, education, income, and BMI

The inclusion of individual level variables into the models did not alter the estimates of the age, period, and cohort effects (Model 3 Additional files 1, 2, and 3). Nevertheless, these variables were independently related to the physical activity outcome variables. Women were less likely to participate in active LTPA (OR = 0.75, 95% CI: 0.70; 0.81) and in active commuting (OR = 0.84, 95% CI: 0.80; 0.89). Likewise, obese individuals were less likely to participate in active LTPA (OR = 0.42, 95% CI: 0.37; 0.47) and in active commuting (OR = 0.82, 95% CI: 0.74; 0.90). In contrast, those with higher education and/or income were more likely to participate in active LTPA and less likely to participate in active commuting (Model 3 in Additional files 1 and 2, respectively). Predictors of sedentary behavior were being female (OR = 1.17, 95% CI: 1.06; 1.29), having higher education (OR = 2.08, 95% CI: 1.67; 2.59) and higher income (OR = 1.45, 95% CI: 1.33; 1.58), and being obese (OR = 1.85, 95% CI: 1.58; 2.17) (Additional file 3, Model 3).

### Relationship between sedentary behavior and physical activity

**Fig. 3:**
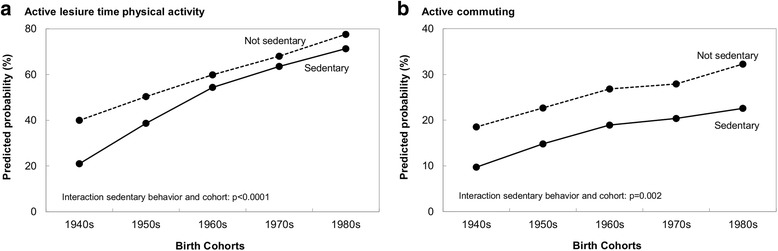
Leisure time physical activity (active) and active commuting by sedentary behavior and birth cohort. Canadian National Population Health Survey, 1994–2011 Birth cohorts: 1980s = Born 1975–84; 1970s = Born 1965–74; 1960s = Born 1955–64; 1950s = Born 1945–55; 1940s = Born 1935–44. Predictions calculated with variables at their average from models in Additional file 4. Each panel shows *p*-values for the interaction between birth cohort and sedentary behavior

The inclusion of sedentary behavior into the active LTPA and active commuting models (Additional file 4) showed that overall those with sedentary behavior were less likely to participate in active LTPA or commuting. Further examinations indicated that there was a significant interaction between sedentary behavior and birth cohort in both, the active LTPA and active commuting models. Differences in active LTPA by sedentary behavior were only significant for those in the 1940s and 1950s cohorts (Fig. 3a), while differences in active commuting by sedentary behavior were seen in all cohorts (Fig. 3b).

### Sensitivity analyses

We made several assumptions while categorizing LTPA and commuting activities. We conducted a series of sensitivity analyses to examine the impact of these choices to the results. The NPHS uses energy expenditure to categorized participants according to their level of LTPA. In the main analyses we have defined active LTPA as those with at least moderate LTPA (≥1.5 kcal/kg/day). For the sensitivity analyses we categorized participants as being active if they engaged in vigorous LTPA (≥3.0 kcal/kg/day). In addition, physical activity guidelines recommend that adults should accumulate at least 150 min of moderate-to-vigorous (in bouts of 10 min) physical activity per week. It has been shown that the definition of active LTPA that we used in this study is somewhat equivalent to the recommended guidelines [28]. However, to test whether our findings were affected by the physical activity definition chosen, for each participant we calculated the total minutes spent in moderate-to-vigorous activities (> 15 min per occasion) per week. We then deemed participants to engage in active LTPA if they achieve the recommended levels of physical activity. We then re-estimated the models for energy expenditure (as a continuous variable), for vigorous LTPA and for meeting physical activity recommendations. Furthermore, in our main analysis we used a cut-off point of 6 or more hours/week of commuting to classify participants as engaging in active commuting. This cut-off point is well above the recommended ≥150 min/week of physical activity; hence we re-estimated the models defining active commuting as reporting 1or more hours per week of biking or walking for transportation. Although the results of these analyses produced different prevalence estimates for active LTPA and active commuting, the patterns by age, period and cohort as well as the effect of sex, education, income, and BMI on the physical activity outcomes did not change our main conclusions.

Given the longitudinal nature of the NPHS, by the end of the study, 29.4% and 4.0% of the baseline sample dropped-out and died, respectively. Participants who died during the course of the study were more likely to be older, men, have lower income and/or education, and to have sedentary behavior, while participants who dropped-out the study were more likely to be younger, men, and have lower income and/or education. No significant differences were seen by BMI and physical activity. As these differences have the potential to bias the findings, we conducted sensitivity analyses to examine the effect of attrition on the results. These analyses were: a) included indicator variables to identify participants who dropped-out or died before the end of the study in all models, and b) fit the models in a restricted sample of individuals with complete data in the nine cycles. The results from these sensitivity analyses did not change the conclusions derived from the main analyses.

The NPHS used a complex sample design, and as such, Statistics Canada recommends that analyses of data coming from this survey use weights to adjust for the unequal probability of sample selection. Unfortunately the methodology used to disentangle age-period-and cohort effects, the HAPC model, cannot incorporate sampling weights. We did, however, fit the two-level models (Model 1) using the sampling weights and findings were not appreciable different to those from the un-weighted analyses.

## Discussion

The major findings from this study are two-fold. First, there is a strong cohort effect in active LTPA, active commuting, and sedentary behavior, such that recent generations are more likely to report being physically active in leisure time and commuting, and at the same time to be more sedentary. Second, underlying these cohort effects is a substantial period effect to both increased participation in physical activity (active LTPA and active commuting) and increased sedentary behavior over time from 1994/95 to 2010/11. The higher participation in active LTPA and active commuting in more recent cohorts was related to this period effect (secular trend) of increasing physical activity over time. Another key finding is that, generally, those with sedentary behavior were less likely to engage in physical activities, particularly in active commuting.

Previous studies have examined changes over time in physical activities [11–16, 23], but the examination of the contribution of age, period, and cohort to changes over time in physical activities across different domains has been more limited [17, 20, 24–26]. The findings of higher participation in active LTPA are in line with some studies [17, 20, 25] but not others [24]. Two studies found that when compared at the same age more recent cohorts were more likely to exercise regularly or to be physically active in leisure time [17, 25]. Whereas another study found that the volume and duration of leisure time physical activity was lower in more recent cohorts of Australians [24]. Our finding of greater participation in active commuting among recent cohorts is in contrast to a study comparing active commuting by birth cohort in the Finish population [17]. One possibility for the discrepancies between our findings for active LTPA and commuting with those in the literature is that our study used longitudinal panel data in which the same individuals were followed over time, while those studies are based on combined data from multiple cross-sectional surveys [17, 24]. Also the Australian study [24] examined average energy expenditure, whereas in our study we examined a binary variable identifying individuals who met recommended activity levels. The greater sedentary behavior in more recent cohorts is in line with the few studies that have compared sedentary behavior by birth cohort [17, 26]. The Finish study used a similar measured as ours and found that sedentary behavior was greater in more recent cohorts [17]. In addition, a longitudinal study examining energy expenditure at work or at home among Chinese adults, found that more recent cohorts have lower levels of physical activity at work or at home [26].

That sedentary behavior has increased over time is in keeping with previous research [15, 17, 19, 23], while there is less agreement about trends over time in LTPA and active commuting. Likewise, the trend of increasing LTPA in more recent years in Canada is in line with studies from Canada [19], Denmark [20], and Finland [17], and in contrast to two studies from Australia [24] and Norway [23]. Likewise, the higher participation in commuting activities is in accord with some studies [19, 22], but not others [17]. The increasing trend in participation in physical activities over time contrasts the increasing trend in obesity in the population. This paradoxical result may be explained by that leisure time and commuting-related activities only account for a small portion of the overall daily energy expenditure by adults, with physical activity at work accounting for most of the energy expenditure [28]. It could also be that the levels of these types of activities are not enough to curb the obesity epidemic and overcome the effects of changes in eating patterns [34, 35]. In addition to individual factors, the influence of physical and social environment on health behaviors is widely recognized [36, 37]. Therefore, it is reasonable to hypothesize that changes in participation in physical activities is influenced by societal changes, such as social norms and/or policies with regards to physical activity over time. It is possible that health promotion efforts and increase awareness of the importance of exercise in the population over time has increased participation in physical activities. Unfortunately, we did not have data on this to test the hypothesis.

As has been found more generally, women were less likely to report participating in physical activities and more likely to be sedentary [38–40]. We also found that those with higher SES (as shown by higher income and education) were more likely to report participating in active LTPA, but not in active commuting. As expected those who were obese were more likely to be sedentary and were also less likely to engage in active LTPA and commuting [41, 42].

The inverse relationship between sedentary behavior and participation in physical activity concurs with a study combining cross-sectional data from 20 countries [43]. However, a further study from Australia suggested the reverse: that sedentary activity was partially compensated by physical activity [44]. The negative impact of modern changes in transportation, occupations and domestic activities on total energy expenditure along with increasing sedentary behavior has been noted [15]. Recent findings also suggest that leisure time, in the context of sedentary lifestyles, is unlikely to be sufficient to prevent increasing population levels of overweight, obesity and chronic disease [34]. In contrast, a recent meta-analysis found that high levels of physical activity attenuated the increased mortality risks associated with sedentary behavior [45]. Furthermore, a prospective population-based study in the UK found that active commuting reduced the risks of cardiovascular diseases, cancer and all-cause mortality [46]. Therefore, it is crucial to adopt a broader approach to understanding and influencing sedentary behavior in addition to increasing physical activity. One possibility is promoting walking or biking for transportation as a way to increase the overall physical activity levels in the population, and particularly for those in more sedentary occupations; and, at a more macro level, the importance of considering city planning, safety and the creation of appealing environments for walking and biking [47, 48]. Perhaps another alternative is to examine the impact of breaking-up prolonged sitting time and how this might relate to increasing light and moderate-to-vigorous intensity physical activities. Studies suggest that there may be metabolic benefits to regularly breaking-up prolonged sitting time in addition to reducing overall sedentary time [49, 50].This is particularly relevant at the workplace, where employers could develop and implement initiatives to improve and maintain the well-being of their workforce.

### Limitations and strengths

An important limitation of this study is that our analyses are based on self-reported data. Studies have shown that self-reported measures are subjected to recall and measurement bias. We do not expect that the patterns seen over time to be affected by recall or measurement bias, as these biases are unlikely to vary over time. In addition, it has been suggested that self-reported physical activity measures may also be affected by social desirability bias [51]. However, a systematic review of the literature comparing objective and self-reported measures for assessing physical activity indicated that studies show inconsistent findings: self-reported measures both over- and under-estimated accelerometer data [52]. Combined data from the Canadian Health Measures Survey (CHMS) from 2007 to 2010 show that estimated minutes/day from the accelerometer and LTPA questionnaire were similar among those 12–59 years, while those 60 years or older over-reported their participation in LTPA [53]. The study also showed overall weak correlations between self-reported and accelerometer LTPA. A comparison with American data showed discrepancies similar to those observed in CHMS [54]. Furthermore, one study found that overweight or obese individuals overestimated energy expenditure from self-reported physical activity questionnaires [55]. Another study comparing self-reported physical activity to accelerometer data found that the accuracy of self-reports was higher for men and for those with a lower BMI [56]. We have somewhat accounted for the potential inconsistencies in self-reported physical activity by BMI and sex, as we have controlled for these variables in the models. Although cross-sectional studies have collected data with accelerometer [52–54], we are not aware of longitudinal panel studies or studies examining age-period-cohort effects with synthetic cohorts (i.e. combining repeated cross-sectional surveys). Given the limited information on changes over time in more objective measures of physical activity, it is difficult to ascertain to what extent changes in self-reported measures reflect real changes in physical activity or if these changes are driven by reporting biases associated with awareness of the importance of physical activity for health. This is an area that merits further research.

In order to examine physical activity trajectories, participants were included if they provided information on at least three cycles of data collection and responded to the physical activity questions at baseline. We found differences related to age, income and BMI between participants included for analysis and those who were excluded. As such, the generalizability of our results may be limited by these differences. Another limitation of the study is the attrition due to drop-outs and mortality, particularly in the older cohort. We re-estimated the models by including indicators variables identifying those who died and those who dropped-out. We also re-estimated the models using the sample of participants with data in the nine cycles. Findings from these analyses did not change our conclusions.

The major strength of this study was the use of data from a large and representative survey of the Canadian population spanning 16 years, which allowed the examination of changes over time independently from the effects of aging and birth cohort. Another strength of the study is that the NPHS collected information on physical activities in three domains: leisure time, commuting, and daily activities, which allowed us to comprehensively examine the patterns of physical activities over the time period.

## Conclusion

This longitudinal study of the population showed increased physical activity levels from 1994 to 2011 in Canada and that this increase was associated with birth cohort differences in participation in physical activity. From a public health perspective monitoring changes over time in physical activity across all domains is important to identify and target groups in the population when developing interventions. In our study, we observed that recent generations reported greater participation in LTPA and in active commuting than their predecessors and that these differences were partially related to broad social and environmental factors that have impacted all ages. Since physical activity and sedentary behavior are risk factors of numerous chronic conditions, interventions to improve and maintain participation in physical activities and reduce sedentary behavior can be targeted to the population as a whole and need not single out particular birth cohorts. In addition, given the increase in sedentary behavior over time and its inverse relationship with participation in physical activity, it is important to develop strategies to balance physical activities and sedentary behavior particularly for those born between 1975 and 1984.

## Additional files

Additional file 1: Results from Logistic Two-level Growth Model (1) and Hierarchical Age-Period-Cohort Models (2 & 3) for Active Leisure Time Physical Activity. Canadian National Population Health Survey, 1994-2011. (DOCX 17 kb)

Additional file 2: Results from Logistic Two-level Growth Model (1) and Hierarchical Age-Period-Cohort Models (2 & 3) for Active Commuting. Canadian National Population Health Survey, 1994-2011. (DOCX 17 kb)

Additional file 3: Results from Logistic Two-level Growth Model (1) and Hierarchical Age-Period-Cohort Models (2 & 3) for Sedentary Behavior. Canadian National Population Health Survey, 1994-2011. (DOCX 17 kb)

Additional file 4: Results from Hierarchical Age-Period-Cohort Models (with Age - Sedentary Behavior and Birth Cohort - Sedentary Behavior Interactions) for Active Leisure Time Physical Activity and Active Commuting. Canadian National Population Health Survey, 1994-2011. (DOCX 18 kb)

## Abbreviations

BMI: Body mass index
CHMS: Canadian health measures survey
HAPC: Hierarchical age-period-cohort
LTPA: Leisure time physical activity
NPHS: National population health survey
