# Supplementary material for: Generational differences in patterns of physical activities over time in the Canadian population: an age-period-cohort analysis
Source: BMC Public Health. 2018 Mar 2;18:304. doi: 10.1186/s12889-018-5189-z (PMC5833083; doi:10.1186/s12889-018-5189-z)
Supplement: Supplementary file 1 — Results from Logistic Two-level Growth Model (1) and Hierarchical Age-Period-Cohort Models (2 & 3) for Active Leisure Time Physical Activity. Canadian National Population Health Survey, 1994-2011. (DOCX 17 kb) [file 12889_2018_5189_MOESM1_ESM.docx]

Results from Logistic Two-level Growth Model (1) and Hierarchical Age-Period-Cohort Models (2 & 3) for Active Leisure Time Physical Activity. Canadian National Population Health Survey, 1994-2011

|  | **MODEL 1** |  | **MODEL 2** |  | **MODEL 3** |
| --- | --- | --- | --- | --- | --- |
|  | OR (95% CI) |  | OR (95% CI) |  | OR (95% CI) |
| **Fixed Effects** |  |  |  |  |  |
| Linear Age^a^ | 1.60 (1.60; 1.61)*^***^* |  | 0.78 (0.77; 0.79)*^***^* |  | 0.72 (0.71; 0.73)*^***^* |
| Birth Cohort (Ref: 1940s) |  |  |  |  |  |
| 1950s | 1.93 (1.63; 2.29)*^***^* |  | 0.93 (0.62; 1.41) |  | 0.92 (0.61; 1.40) |
| 1960s | 3.54 (3.02; 4.15)*^***^* |  | 0.84 (0.58; 1.20) |  | 0.78 (0.54; 1.12) |
| 1970s | 6.52 (5.60; 7.60)*^***^* |  | 0.77 (0.56; 1.06) |  | 0.65 (0.47; 0.89)*^**^* |
| 1980s | 12.96 (10.82; 15.52)*^***^* |  | 0.75 (0.47; 1.20) |  | 0.57 (0.36; 0.91)*^**^* |
| Sex (Women) |  |  |  |  | 0.75 (0.70; 0.81)*^**^* |
| Education  (Ref: <12 years) |  |  |  |  |  |
| 16+ years |  |  |  |  | 1.57 (1.33; 1.84)*^***^* |
| 12-15 years |  |  |  |  | 1.20 (1.12; 1.29)*^***^* |
| Income Quartiles  (Ref: Bottom (Q1)) |  |  |  |  |  |
| Top (Q4) |  |  |  |  | 1.42 (1.33; 1.51)*^***^* |
| Q3 |  |  |  |  | 1.18 (1.11; 1.25)*^***^* |
| Q2 |  |  |  |  | 1.08 (1.02; 1.15)*^**^* |
| Non-response |  |  |  |  | 0.97 (0.85; 1.11) |
| BMI (Ref: Normal)^b^ |  |  |  |  |  |
| Severe Obese |  |  |  |  | 0.42 (0.37; 0.47)*^***^* |
| Moderate Obese |  |  |  |  | 0.64 (0.59; 0.69)*^***^* |
| Overweight |  |  |  |  | 0.86 (0.82; 0.91)*^***^* |
| Underweight |  |  |  |  | 0.91 (0.79; 1.04) |
| **Random Effects**^c^ |  |  |  |  |  |
| Individual | 1.53 (1.49; 1.56)*^***^* |  | 1.47 (1.43; 1.50)*^***^* |  | 1.45 (1.42; 1.49)*^***^* |
| Period |  |  | 0.41 (0.21; 0.61)*^***^* |  | 0.42 (0.22; 0.62)*^***^* |

Abbreviations: BMI, Body Mass Index; OR, Odd Ratios; 95% CI, 95% Confidence Interval.

*^***^ p<0.0001, ^**^ p<0.01, ^*^ p<0.05, ^†^ p<0.1*.

^a^ Age was centered at the mean of the age distribution in 1994/95 (35 years). ORs represent increments in 10 years. Models also included a quadratic age term.

^b^ Severe obese (>=35.0), Moderate Obese (30.0-34.9), Overweight (25.0-29.9), Underweight (<18.5), Normal (18.5-24.9).

^c^ Estimates are variance and 95% confidence intervals.
