# Supplementary material for: Generational differences in patterns of physical activities over time in the Canadian population: an age-period-cohort analysis
Source: BMC Public Health. 2018 Mar 2;18:304. doi: 10.1186/s12889-018-5189-z (PMC5833083; doi:10.1186/s12889-018-5189-z)
Supplement: Supplementary file 2 — Results from Logistic Two-level Growth Model (1) and Hierarchical Age-Period-Cohort Models (2 & 3) for Active Commuting. Canadian National Population Health Survey, 1994-2011. (DOCX 17 kb) [file 12889_2018_5189_MOESM2_ESM.docx]

Results from Logistic Two-level Growth Model (1) and Hierarchical Age-Period-Cohort Models (2 & 3) for Active Commuting. Canadian National Population Health Survey, 1994-2011

|  | **MODEL 1** |  | **MODEL 2** |  | **MODEL 3** |
| --- | --- | --- | --- | --- | --- |
|  | OR (95% CI) |  | OR (95% CI) |  | OR (95% CI) |
| **Fixed Effects** |  |  |  |  |  |
| Linear Age^a^ | 0.99 (0.97; 1.01) |  | 0.80 (0.80; 0.81)*^***^* |  | 0.87 (0.86; 0.88)*^***^* |
| Birth Cohort (Ref: 1940s) |  |  |  |  |  |
| 1950s | 1.22 (1.07; 1.40)*^***^* |  | 1.10 (0.82; 1.47) |  | 1.08 (0.80; 1.47) |
| 1960s | 1.54 (1.36; 1.74)*^***^* |  | 1.14 (0.88; 1.48) |  | 1.15 (0.88; 1.51) |
| 1970s | 1.60 (1.43; 1.80)*^***^* |  | 0.89 (0.71; 1.12) |  | 1.01 (0.80; 1.27) |
| 1980s | 1.83 (1.58; 2.12)*^***^* |  | 0.79 (0.57; 1.10) |  | 0.93 (0.66; 1.32) |
| Sex (Women) |  |  |  |  | 0.84 (0.80; 0.89)*^***^* |
| Education  (Ref: <12 years) |  |  |  |  |  |
| 16+ years |  |  |  |  | 0.72 (0.63; 0.82)*^***^* |
| 12-15 years |  |  |  |  | 0.91 (0.86; 0.97)*^***^* |
| Income Quartiles  (Ref: Bottom (Q1)) |  |  |  |  |  |
| Top (Q4) |  |  |  |  | 0.90 (0.85; 0.96)*^***^* |
| Q3 |  |  |  |  | 0.92 (0.87; 0.97)*^***^* |
| Q2 |  |  |  |  | 0.98 (0.93; 1.03) |
| Non-response |  |  |  |  | 1.10 (0.98; 1.24) |
| BMI (Ref: Normal)^b^ |  |  |  |  |  |
| Severe Obese |  |  |  |  | 0.82 (0.74; 0.90)*^***^* |
| Moderate Obese |  |  |  |  | 0.96 (0.90; 1.03) |
| Overweight |  |  |  |  | 0.96 (0.92; 1.01) |
| Underweight |  |  |  |  | 1.07 (0.95; 1.21) |
| **Random Effects**^c^ |  |  |  |  |  |
| Individual | 0.62 (0.58; 0.66)*^***^* |  | 0.82 (0.80; 0.85)*^***^* |  | 0.83 (0.80; 0.86)*^***^* |
| Period |  |  | 0.19 (0.10; 0.29)*^***^* |  | 0.13 (0.06; 0.21)*^***^* |

Abbreviations: BMI, Body Mass Index; OR, Odd Ratios; 95% CI, 95% Confidence Interval.

*^***^ p<0.0001, ^**^ p<0.01, ^*^ p<0.05, ^†^ p<0.1*.

^a^ Age was centered at the mean of the age distribution in 1994/95 (35 years). ORs represent increments in 10 years. Models also included a quadratic age term.

^b^ Severe obese (>=35.0), Moderate Obese (30.0-34.9), Overweight (25.0-29.9), Underweight (<18.5), Normal (18.5-24.9).

^c^ Estimates are variance and 95% confidence intervals.
