# Supplementary material for: Generational differences in patterns of physical activities over time in the Canadian population: an age-period-cohort analysis
Source: BMC Public Health. 2018 Mar 2;18:304. doi: 10.1186/s12889-018-5189-z (PMC5833083; doi:10.1186/s12889-018-5189-z)
Supplement: Supplementary file 3 — Results from Logistic Two-level Growth Model (1) and Hierarchical Age-Period-Cohort Models (2 & 3) for Sedentary Behavior. Canadian National Population Health Survey, 1994-2011. (DOCX 17 kb) [file 12889_2018_5189_MOESM3_ESM.docx]

Results from Logistic Two-level Growth Model (1) and Hierarchical Age-Period-Cohort Models (2 & 3) for Sedentary Behavior. Canadian National Population Health Survey, 1994-2011

|  | **MODEL 1** |  | **MODEL 2** |  | **MODEL 3** |
| --- | --- | --- | --- | --- | --- |
|  | OR (95% CI) |  | OR (95% CI) |  | OR (95% CI) |
| **Fixed Effects** |  |  |  |  |  |
| Linear Age^a^ | 1.24 (1.19; 1.28) *^***^* |  | 1.00 (0.99; 1.01) |  | 0.82 (0.81; 0.82)*^***^* |
| Birth Cohort (Ref: 1940s) |  |  |  |  |  |
| 1950s | 1.29 (1.04; 1.59) *^**^* |  | 0.81 (0.61; 1.07) |  | 0.78 (0.59; 1.03) |
| 1960s | 1.61 (1.32; 1.97) *^***^* |  | 0.60 (0.46; 0.78) *^***^* |  | 0.62 (0.48; 0.80)*^***^* |
| 1970s | 2.20 (1.81; 2.68) *^***^* |  | 0.55 (0.43; 0.71) *^***^* |  | 0.58 (0.45; 0.74)*^***^* |
| 1980s | 4.67 (3.72; 5.86) *^***^* |  | 1.00 (0.74; 1.35) |  | 1.02 (0.75; 1.39) |
| Sex (Women) |  |  |  |  | 1.17 (1.06; 1.29)*^***^* |
| Education  (Ref: <12 years) |  |  |  |  |  |
| 16+ years |  |  |  |  | 2.08 (1.67; 2.59)*^***^* |
| 12-15 years |  |  |  |  | 0.92 (0.84; 1.02) |
| Income Quartiles  (Ref: Bottom (Q1)) |  |  |  |  |  |
| Top (Q4) |  |  |  |  | 1.45 (1.33; 1.58)*^***^* |
| Q3 |  |  |  |  | 1.15 (1.07; 1.25)*^***^* |
| Q2 |  |  |  |  | 1.04 (0.96; 1.12) |
| Non-response |  |  |  |  | 0.90 (0.75; 1.10) |
| BMI (Ref: Normal)^b^ |  |  |  |  |  |
| Severe Obese |  |  |  |  | 1.85 (1.58; 2.17)*^***^* |
| Moderate Obese |  |  |  |  | 1.13 (1.02; 1.26)*^***^* |
| Overweight |  |  |  |  | 1.03 (0.96; 1.11) |
| Underweight |  |  |  |  | 1.42 (1.20; 1.67)*^***^* |
| **Random Effects**^c^ |  |  |  |  |  |
| Individual | 1.95 (1.90; 2.00)*^***^* |  | 1.77 (1.71; 1.84) *^***^* |  | 1.75 (1.68; 1.81)*^***^* |
| Period |  |  | 0.30 (0.29; 0.32) *^***^* |  | 0.29 (0.28; 0.31)*^***^* |

Abbreviations: BMI, Body Mass Index; OR, Odd Ratios; 95% CI, 95% Confidence Interval.

*^***^ p<0.0001, ^**^ p<0.01, ^*^ p<0.05, ^†^ p<0.1*.

^a^ Age was centered at the mean of the age distribution in 1994/95 (35 years). ORs represent increments in 10 years. Models also included a quadratic age term.

^b^ Severe obese (>=35.0), Moderate Obese (30.0-34.9), Overweight (25.0-29.9), Underweight (<18.5), Normal (18.5-24.9).

^c^ Estimates are variance and 95% confidence intervals.
