# Supplementary material for: Generational differences in patterns of physical activities over time in the Canadian population: an age-period-cohort analysis
Source: BMC Public Health. 2018 Mar 2;18:304. doi: 10.1186/s12889-018-5189-z (PMC5833083; doi:10.1186/s12889-018-5189-z)
Supplement: Supplementary file 4 — Results from Hierarchical Age-Period-Cohort Models (with Age - Sedentary Behavior and Birth Cohort - Sedentary Behavior Interactions) for Active Leisure Time Physical Activity and Active Commuting. Canadian National Population Health Survey, 1994-2011. (DOCX 18 kb) [file 12889_2018_5189_MOESM4_ESM.docx]

Results from Hierarchical Age-Period-Cohort Models (with Age - Sedentary Behavior and Birth Cohort - Sedentary Behavior Interactions) for Active Leisure Time Physical Activity and Active Commuting. Canadian National Population Health Survey, 1994-2011

|  | **MODERATE-TO-VIGOROUS PHYSICAL ACTIVITY** | |  | **ACTIVE**  **COMMUTING** | |
| --- | --- | --- | --- | --- | --- |
|  | Estimate | S.E. |  | Estimate | S.E. |
| Linear Age^a^ | -0.043*^***^* | 0.002 |  | 0.003 | 0.002 |
| Birth Cohort (Ref: 1940s) |  |  |  |  |  |
| 1950s | 0.420*^***^* | 0.059 |  | 0.253*^***^* | 0.056 |
| 1960s | 0.806*^***^* | 0.068 |  | 0.476*^***^* | 0.069 |
| 1970s | 1.156*^***^* | 0.081 |  | 0.532*^***^* | 0.083 |
| 1980s | 1.647*^***^* | 0.097 |  | 0.738*^***^* | 0.100 |
| Sex (Women) | -0.173*^***^* | 0.031 |  | -0.016 | 0.026 |
| Education (Ref: <12 years) |  |  |  |  |  |
| 16+ years | 0.550*^***^* | 0.044 |  | 0.100*^*^* | 0.041 |
| 12-15 years | 0.208*^***^* | 0.033 |  | 0.060*^†^* | 0.031 |
| Income Quartiles  (Ref: Bottom (Q1)) |  |  |  |  |  |
| Top (Q4) | 0.428*^***^* | 0.034 |  | -0.092*^*^* | 0.036 |
| Q3 | 0.203*^***^* | 0.029 |  | -0.031 | 0.032 |
| Q2 | 0.090*^**^* | 0.028 |  | -0.102 | 0.033 |
| Non-response | 0.013 | 0.064 |  | -0.125*^†^* | 0.073 |
| BMI (Ref: Normal)^b^ |  |  |  |  |  |
| Severe Obese | -0.673*^***^* | 0.055 |  | -0.181*^**^* | 0.057 |
| Moderate Obese | -0.316*^***^* | 0.036 |  | -0.002 | 0.037 |
| Overweight | -0.084*^**^* | 0.0250 |  | 0.004 | 0.027 |
| Underweight | -0.130*^*^* | 0.065 |  | 0.018 | 0.070 |
| Sedentary behavior | -0.891*^***^* | 0.066 |  | -0.758*^***^* | 0.086 |
| Sedentary behavior by Age | -0.003 | 0.003 |  | 0.002 | 0.004 |
| Sedentary behavior by Birth Cohort (Ref: 1940s) |  |  |  |  |  |
| 1950s | 0.443*^***^* | 0.084 |  | 0.258*^*^* | 0.104 |
| 1960s | 0.694*^***^* | 0.083 |  | 0.332*^**^* | 0.104 |
| 1970s | 0.721*^***^* | 0.083 |  | 0.295*^**^* | 0.101 |
| 1980s | 0.586*^***^* | 0.085 |  | 0.224*^*^* | 0.106 |
| Random Effects^c^ |  |  |  |  |  |
| Individual | 1.450*^***^* | 0.018 |  | 0.781*^***^* | 0.015 |
| Period | 0.420*^***^* | 0.104 |  | 0.132*^***^* | 0.039 |

Abbreviations: BMI, Body Mass Index. *^***^ p<0.0001, ^**^ p<0.01, ^*^ p<0.05, ^†^ p<0.1*.

^a^ Age was centered at the mean of the age distribution in 1994/95 (35 years). Models also included a quadratic age term.

^b^ Severe obese (>=35.0), Moderate Obese (30.0-34.9), Overweight (25.0-29.9), Underweight (<18.5), Normal (18.5-24.9).

^c^ Estimates are variance and 95% confidence intervals.
